# The Impact of Psychological Resilience on Digital Transformation: A Resource Orchestration Perspective

**DOI:** 10.3390/bs16081340

**Published:** 2026-08-04

**Authors:** Gejun Wang, Xingqiu Hu

**Affiliations:** School of Business, Hohai University, Nanjing 211100, China; huxq@hhu.edu.cn

**Keywords:** executive psychological resilience, enterprise digital transformation, resource orchestration theory, too-much-of-a-good-thing effect, managerial discretion

## Abstract

In the context of economic turbulence, pursuing new development opportunities through digital transformation has become an inevitable choice for Chinese firms, with top executives serving as core decision-makers. Drawing on resource orchestration theory and the too-much-of-a-good-thing effect, this study examines the relationship between executive psychological resilience and enterprise digital transformation, as well as its boundary conditions, using panel data from Chinese A-share listed firms from 2011 to 2024. The results provide evidence of an inverted U-shaped relationship between executive psychological resilience and enterprise digital transformation, suggesting that an optimal level of psychological resilience exists for digital transformation. Beyond this level, manifestations of bounded rationality, including an underestimation of transformation risks and attentional biases, lead to inefficient resource orchestration. Industry capital intensity weakens this nonlinear relationship, while slack resources appear to shift the turning point to an earlier stage. By questioning the cognitive inertia that treats resilience as a universally beneficial personality trait, this study shifts the perspective on the relationship between executive psychological resilience and enterprise digital transformation from a linear to a nonlinear framework. It further extends the application of the resource orchestration theory in the digital transformation context and contributes to research on the micro-level drivers of digital transformation—specifically executive psychological traits.

## 1. Introduction

Enterprise digital transformation (DT) refers to the systematic reconfiguration and upgrading of firms’ value creation logic enabled by digital technologies. It reflects a micro-level manifestation of the deep integration between the digital economy and the real economy and serves as a key pathway for firms to achieve high-quality development. Vial (2019) reviewed 282 relevant studies and developed a comprehensive framework of digital transformation. He conceptualized DT as a dynamic and cyclical process characterized by technology-triggered disruptions, strategy-driven actions, structural change and organizational constraints, leading to a range of subsequent organizational and environmental outcomes. Prior research documented that digital transformation generates a broad set of positive organizational outcomes, highlighting the importance of examining its antecedents from both theoretical and practical perspectives. Existing research has emphasized the central role of top management in shaping firms’ digital transformation trajectories. DT is typically initiated by managerial intent and professional competence, which shape executives’ cognition regarding its essence, strategic vision, and implementation pathways. Successful transformation further depends on executives’ digital capabilities and change-management skills, as well as dynamic managerial capabilities such as fostering decentralized structures and cultivating a risk-tolerant organizational culture (Sousa-Zomer et al., 2020; Wrede et al., 2020). Bilal et al. (2024) further show that transformation failures in many firms stem from executives’ narrow view of digital transformation as a purely technological upgrade, rather than as a comprehensive strategic and organizational transformation. However, digital transformation unfolds in a highly turbulent environment shaped by rapid technological change. Firms face multiple challenges, including organizational inertia, employee resistance, and emerging ethical concerns related to data security and privacy, creating a complex multi-stakeholder environment characterized by diverse pressures and elevated failure risks.

Psychological resilience (PR) refers to an individual’s response process in the face of adversity (Ahmed et al., 2022). Executive psychological resilience thus represents a critical psychological resource that enables executives to cope with pressure and promote firm development in an uncertain environment. In 2018, Huawei faced comprehensive technological sanctions imposed by the United States. Under the leadership of Ren Zhengfei, the top management team demonstrated strong psychological resilience and guided the firm through strategic restructuring, mobilizing organizational resources to sustain development under extreme external pressure. Similarly, following the implementation of China’s “Double Reduction” policy, Yu Minhong and his core management team relied on strong self-regulatory capabilities to transform their business and successfully launch the Oriental Selection livestreaming platform. These cases underscore the critical role of executive psychological resilience in enabling firms to navigate external shocks and accomplish organizational transformation under adverse conditions. However, according to Accenture’s 2024 China Enterprise Digital Transformation Index Report, only around 30% of Chinese firms believe they are adequately prepared to cope with changes related to geopolitics, environmental challenges, and economic uncertainty—more than 10 percentage points lower than the global average. Upper echelons theory builds on the concept of bounded rationality. Existing research has mainly examined how observable managerial characteristics, such as educational background, career experience, digital leadership, and digital literacy, shape executives’ cognitive bases and values. These characteristics influence how executives process information, perceive problems, and ultimately make strategic choices. However, these variables do not capture a critical dimension of executives’ ability to withstand pressure and recover from adversity. Singh and Hess (2020) found that in the context of digital transformation, executives must possess a high level of resilience and perseverance, as well as the ability to anticipate resistance from colleagues and other stakeholders and confront the related challenges. Although prior research has predominantly emphasized the positive outcomes of resilience, recent studies suggest that higher psychological resilience is not necessarily more beneficial (Williams et al., 2017). Hallak et al. (2018) pointed out that entrepreneurial resilience does not directly improve firm performance. Rather, it influences performance indirectly by enhancing creative self-efficacy and innovative behavior, suggesting that resilience should not be viewed as a universally positive trait. When resilience is excessively high, it may induce psychological states such as false hope syndrome, narcissism, and cognitive bias, thereby impairing individuals’ ability to accurately assess environmental risks (Fu et al., 2023; Hartmann et al., 2022; Jiao et al., 2024; Mahdiani & Ungar, 2021). Tlaiss and Khanin (2025), based on evidence from Lebanese female entrepreneurs, further pointed out that persistent resilience may lead to entrepreneurial burnout and escalation of commitment. Ignoring the “dark side” of resilience reflects an overly idealized assumption that executives always identify optimal coping strategies and achieve growth through adversity. This assumption is inconsistent with bounded rationality and may explain the mixed findings in the existing literature on executive psychological resilience.

Overall, existing research reveals two research directions that remain to be further explored. First, although executive psychological resilience has been widely studied, its potential nonlinear effects remain insufficiently explored. By investigating the “too-much-of-a-good-thing” effect of executive resilience, this study shifts the research perspective on the relationship between resilience and digital transformation from a linear to a non-linear paradigm, thereby challenging the prevailing assumption that resilience is an inherently beneficial trait. Second, existing studies grounded in upper echelons theory have highlighted the central role of executives in enterprise digital transformation. Although upper echelons theory effectively explains how executives’ explicit individual characteristics manifest at the firm level, it provides limited insight into how implicit psychological mechanisms influence firm behavior. To fill this theoretical gap, we introduce resource orchestration theory (ROT) to provide a process-oriented explanation of how executives respond to pressure in the resource orchestration process. This extension helps to better capture and clarify the functioning process of resilience.

## 2. Theoretical Analysis and Hypotheses

### 2.1. Resource Orchestration Theory

Sirmon et al. (2010) proposed resource orchestration theory (ROT) by integrating the two complementary and interrelated frameworks: resource management and asset orchestration. The theory argues that merely possessing resources that are valuable, rare, inimitable, and non-substitutable (VRIN) is insufficient to generate competitive advantage. Instead, competitive advantage depends on managers’ actions in managing and orchestrating resources. The resource management framework involves structuring the resource portfolio (acquiring, accumulating, and divesting resources), bundling resources to build capabilities (stabilizing, enriching, and pioneering), and leveraging capabilities in the marketplace (mobilizing, coordinating, and deploying) to create value. The asset orchestration framework includes two dimensions: search/selection and configuration/deployment. The search/selection process requires managers to identify and invest in assets, design organizational and governance structures, and develop business models. The configuration/deployment process requires the coordination of complementary assets, vision planning, and innovation support. The integration of these two frameworks forms a comprehensive resource orchestration perspective.

The core of resource orchestration theory lies in the enactment of managerial cognition. In the digital context, resource structuring enables firms to incorporate emerging digital technologies into their existing resource portfolios, thereby facilitating digital transformation. Resource bundling emphasizes the process of combining digital assets with knowledge to develop digital capabilities, ultimately creating value for both customers and owners. Finally, resource leveraging refers to embedding digital technologies into business processes and models to realize value creation. Resource orchestration theory has been widely employed as a primary theoretical lens for investigating firms’ ambidextrous innovation (Jiang et al., 2025) and green technology innovation efficiency (Zhao et al., 2025). Furthermore, an increasing number of studies have applied this framework to explore the relationship between executive characteristics and enterprise digital transformation (Lee et al., 2026; Wei et al., 2025; Wu et al., 2025).

### 2.2. The “Too-Much-of-a-Good-Thing” Effect of Executive Psychological Resilience

#### 2.2.1. Research on Executive Psychological Resilience

The concept of resilience originated in the field of physics, where it initially referred to the property of elasticity. It was subsequently introduced into medicine and psychology, with early research primarily focusing on adolescents and vulnerable children. Existing studies indicate that both social environmental factors and individual capability factors influence the formation of executive psychological resilience (Bai, 2025; Elshaer & Saad, 2021). Regarding the nature of psychological resilience, three independent perspectives have emerged: the trait perspective, the process perspective, and the outcome perspective. (a) The trait perspective conceptualizes resilience as a relatively stable individual characteristic reflecting resistance to adversity and the speed of recovery following disruption. For instance, Cooper et al. (2013) identify four core components of workplace resilience: confidence, goal orientation, adaptability, and social support. (b) The process perspective defines managerial resilience as a dynamic capability through which individuals anticipate potential threats, respond effectively to disruptions, adapt to changing conditions, and achieve self-transcendence. This capability develops over time through accumulated experience and is jointly shaped by both individual attributes and contextual conditions (Duchek, 2017; Moenkemeyer et al., 2012). (c) The outcome perspective conceptualizes resilience as an outcome, defined as a state in which the degree of disruption to an individual’s psychological functioning following exposure to adversity is lower than expected within a given cultural context. It also refers to a state in which individuals are able to maintain or restore a high level of psychological well-being (Troy et al., 2023).

The field of management has extensively integrated the concept of psychological resilience to examine how individual traits affect firms’ viability and operating performance in the face of crises. Prior studies show that entrepreneurs with higher levels of psychological resilience in small and medium-sized enterprises are more likely to view the COVID-19 pandemic as a surmountable challenge. They also demonstrate greater efficiency in leading their firms, thereby increasing the probability of survival during the pandemic (Chhatwani et al., 2022; Islam et al., 2020). In more routine contexts, existing studies have revealed the mechanisms through which entrepreneurial psychological resilience influences both firm-level and individual-level performance from various theoretical perspectives. Khan et al. (2023) found that entrepreneurial psychological resilience plays a critical mediating role in enhancing firm performance and organizational resilience by promoting adaptive behavior and resource integration. Based on social cognitive theory, Nautiyal and Pathak (2024) further found that entrepreneurial psychological resilience contributes to individual success in career development, financial outcomes, and job satisfaction. They also found that resilience-related factors such as social support, adaptability, sense of purpose, and self-efficacy positively influence firm performance. Drawing on the broaden-and-build theory of positive psychology, Aust et al. (2024) argued that entrepreneurial psychological resilience is reflected in how entrepreneurs interpret failure and explain setbacks, as well as how they reframe adverse events as learning processes, temporary deviations, or necessary costs in the pursuit of higher goals. This perspective reveals that entrepreneurial psychological resilience is a cognitive reinterpretation capability rather than merely tolerance for stress.

#### 2.2.2. A Mechanism Explanation Based on the “Motivation–Opportunity Interaction”

Grant and Schwartz (2011) first proposed the “too-much-of-a-good-thing (TMGT) effect”, suggesting that variables typically regarded as beneficial possess a threshold, beyond which their positive relationship between such a variable and the outcome variable ceases and becomes negative, resulting in an overall inverted U-shaped nonlinear relationship. Fundamentally, the “too-much-of-a-good-thing effect” operates through two distinct mechanisms. The first is the additive effect of benefit/cost, in which benefits represent beneficial mechanisms through which changes in the independent variable increase the dependent variable, whereas costs refer to another mechanism through which changes in the independent variable reduce the dependent variable. The net effect is calculated through a simple algebraic summation of these two mechanisms, making it the most common explanation for the too-much-of-a-good-thing effect. The second category is the motivation/ability (or opportunity) interaction mechanism (Haans et al., 2015). In this context, motivation refers to individuals’ subjective willingness to engage in a specific behavior, while ability reflects the corresponding psychological and cognitive capabilities. Opportunity denotes the relevant environmental factors. The core tenet is that an increase in the independent variable typically triggers a trade-off between the individual’s motivation and their ability (or opportunity) to perform a given behavior. These two types of mechanisms mutually moderate each other’s effects, and their interaction is reflected in the total effect through a multiplicative relationship.

The effectiveness of digital transformation depends largely on executives’ ability to dynamically allocate and orchestrate resources in response to both internal and external environmental changes. Resource structuring involves resource acquisition, accumulation, and divestment. Firms acquire digital resources from the market, such as big data, artificial intelligence, blockchain, and cloud computing technologies. When the digital factor market cannot fully satisfy resource demands, firms must develop and accumulate internal resources while evaluating existing resources and divesting those with low strategic value (Cheng et al., 2023). This process also includes the development of digital talent and the accumulation of digital assets. In this stage, failed resource acquisition and short-term performance declines may trigger resistance from stakeholders, such as shareholders and employees. In the face of such adversity, resilient executives exhibit a greater tolerance for uncertainty and remain committed to the long-term strategic objectives of digital transformation. Consequently, they are more likely to embrace rather than resist change, helping firms successfully navigate the exploration stage of digital transformation. Resource bundling consists of stabilizing, enriching, and pioneering activities. This process may face conflicts between technology departments and existing business units, as well as difficulties in shifting from legacy operational and production processes to digitally enabled ones. Under such circumstances, resilient executives are more likely to actively promote cross-functional resource integration, adjust organizational structures and processes, and foster a culture conducive to digital transformation. Furthermore, the success of resource bundling depends not only on the development of internal capabilities but also, to a large extent, on collaboration with external partners across the supply chain to achieve complementary resource advantages. Resilient executives are more likely to leverage their personal networks and reputational capital to establish cooperative relationships that further enhance digital transformation. Resource leveraging involves mobilizing, coordinating and deploying to create value for customers and owners. At this stage, executives must identify and deploy the capabilities necessary to exploit market opportunities. This requires executives to coordinate efforts across organizational units. It also requires effectively combining resources and capabilities to support business model renewal and the digital transformation of production systems and marketing.

Resource orchestration is essentially a process of allocating managerial cognition and attention. However, human rationality is very approximate in the face of the complexities of organizational life (Simon, 1991). Bounded rationality prevents executives from fully processing available information, making resource orchestration outcomes dependent on their attentional focus and behavioral tendencies. First, excessively resilient executives may be unable to accurately assess the obstacles and tend to filter negative feedback associated with digital investments (Fu et al., 2023; Hartmann et al., 2022). This may lead firms to undertake digital investments whose complexity exceeds their resource orchestration capacity. Such misallocation not only introduces substantial resource redundancy but also impedes the divestment of irrelevant legacy resources. Second, the human brain is severely constrained in its computational power, information processing capacity, and attentional resources. The attention-based view argues that organizational decision-makers’ actions and decisions are shaped by the issues and answers to which they attend. Decision-makers, including CEOs, may deviate from purely rational behavior due to cognitive limitations (Ocasio, 1997; Xu et al., 2025). Digital resource orchestration typically involves multiple subprojects such as information system construction, intelligent production process transformation, and customer management digitalization. The simultaneous implementation of these projects places substantial demands on managerial attention. Executives with excessively high levels of psychological resilience may devote disproportionate attention to projects experiencing difficulties rather than prioritizing projects according to their strategic importance and interdependence. As a result, resource allocation becomes fragmented, reducing the efficiency of resource orchestration (S. Jin et al., 2024). Moreover, excessive resilience may impair executives’ capacity for reflection and meaning-making following failure (L. Jin & Xu, 2025). When resource orchestration fails, executives inevitably develop self-assessments of both the economic and psychological costs incurred. Individuals with moderate levels of resilience are better able to leverage their self-regulatory capabilities in such situations. By contrast, excessively resilient executives may become more sensitive to failure and therefore incur greater psychological costs. Prior research suggests that excessive psychological costs hinder constructive learning and personal growth, thereby reducing executives’ willingness and ability to correct ineffective behaviors. At the same time, evaluations of losses may trap them in sunk-cost considerations, making them less likely to reflect on failure and reconfigure resources in a timely manner (Shepherd et al., 2009). Consequently, excessive resilience substantially weakens the conversion of motivation into effective action.

**Figure 1:**
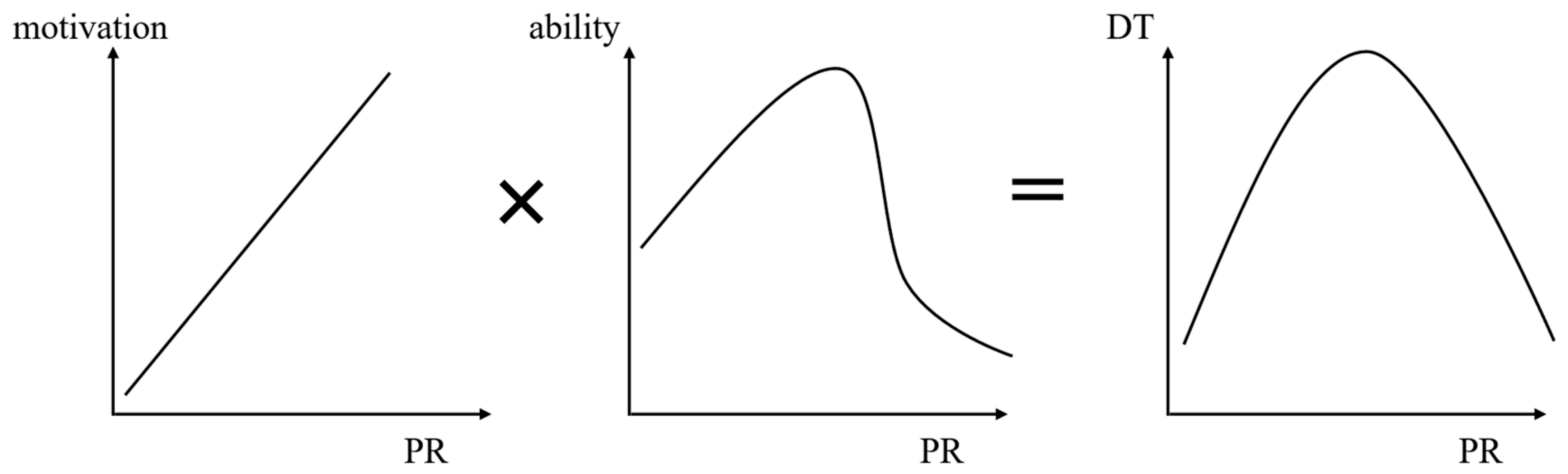
Latent functions resulting in a theoretical inverted U-shaped relationship.

Based on the above analysis, increases in the antecedent variable—executive psychological resilience—lead to divergent shifts in the motivation and ability to drive digital transformation, resulting in a countervailing effect. As a result, the relationship between executive resilience and digital transformation exhibits an inverted U shape. (as shown in Figure 1). Accordingly, we propose:

**Hypothesis** **1.** *There is an inverted U-shaped relationship between executive psychological resilience and enterprise digital transformation.*

### 2.3. The Moderating Effect of Managerial Discretion

Resource orchestration for digital transformation is inherently context-dependent, and executives’ strategic behaviors are profoundly shaped by the firm’s internal and external resource environments. The concept of managerial discretion, introduced by Finkelstein and Hambrick (1989), refers to the latitude of action or range of strategic choices available to executives within a given context. This perspective was developed to reconcile the tension between organizational ecology, which emphasizes environmental and organizational constraints on managerial actions, and strategic choice theory, which highlights the role of executives in shaping organizational outcomes. As a critical contextual condition, managerial discretion determines the extent to which executives’ characteristics, preferences, and behaviors can be translated into organizational outcomes. In setting characterized by high levels of managerial discretion, TMTs’ operations are relatively unconstrained, but in settings where managerial discretion is low, organizational and national conditions impose constraints that limit the TMT’s strategic actions (Saeed et al., 2024; Wangrow et al., 2015). Prior studies have examined this contingency in various settings, including the effects of CEO overconfidence on corporate internationalization and investment efficiency, as well as the relationship between TMT experience and strategic change.

Building on this perspective, we argue that a more complete understanding of how executive resilience affects enterprise digital transformation requires considering the contextual factors that facilitate or constrain executives’ discretion in orchestrating digital resources. Accordingly, we examine moderators across two levels of analysis—environmental and organizational. Specifically, we focus on industrial capital intensity as an environmental-level factor and slack resources as an organizational-level factor. Environmental discretion refers to the latitude of action available to top executives in formulating and implementing strategic decisions within the firm’s external task environment. Industrial capital intensity reflects the level of capital required to establish and sustain operations within a particular industry. Firms operating in capital-intensive industries are often locked into existing strategic paths due to substantial investments in fixed assets, which create strong path dependence and constrain current strategic choices through previous resource commitments (Datta & Rajagopalan, 1998). Consequently, these industries are characterized by high sunk costs, significant risks, and substantial entry barriers. Industrial capital intensity may shape the relationship between executive psychological resilience and digital transformation in two distinct ways. On the one hand, high capital intensity increases organizational rigidity and reduces managerial discretion by limiting executives’ flexibility in reallocating resources and adjusting strategic directions. As a result, executives’ personal characteristics and preferences are less likely to be translated into digital transformation outcomes, thereby weakening the inverted U-shaped relationship between executive psychological resilience and digital transformation (Chen et al., 2025). On the other hand, digital transformation in capital-intensive industries typically requires substantial financial investment and the integration of complex technologies. Resource acquisition and orchestration are constrained by high sunk costs and technological barriers. Under such conditions, when executive psychological resilience exceeds a moderate level, excessive persistence may evolve into an escalation of commitment to existing digital transformation paths rather than a rational adjustment of strategic decisions. This tendency amplifies the negative effect of excessive psychological resilience on digital transformation. Based on the above arguments, we propose the following hypothesis:

**Hypothesis** **2.** *Environmental discretion moderates the inverted U-shaped relationship between executive psychological resilience and enterprise digital transformation.*

Organizational discretion refers to the latitude of action available to executives when formulating and implementing strategic decisions within the internal organizational environment. According to the resource-based view, slack resources represent a pool of discretionary assets that can be allocated to uncertain projects. This study focuses on unabsorbed and potential slack resources, which are relatively flexible and easily redeployed, providing an important material foundation for organizations to adapt to environmental changes and adjust strategic orientations. Firms with abundant slack resources can rapidly invest in new digital technologies without engaging in excessively cautious resource orchestration, thereby improving the efficiency of digital resource orchestration (Heubeck & Ahrens, 2024). Compared with resource-constrained firms, organizations possessing greater slack resources are better able to alleviate goal conflicts, lower the barriers to adopting digital initiatives, and tolerate delayed or uncertain returns from digital projects. Consequently, abundant slack resources provide executives with greater flexibility in allocating and orchestrating digital resources, thereby facilitating the influence of executive psychological resilience on enterprise digital transformation. Therefore, we propose:

**Hypothesis** **3.** *Organizational discretion positively moderates the inverted U-shaped relationship between executive psychological resilience and enterprise digital transformation.*

**Figure 2:**
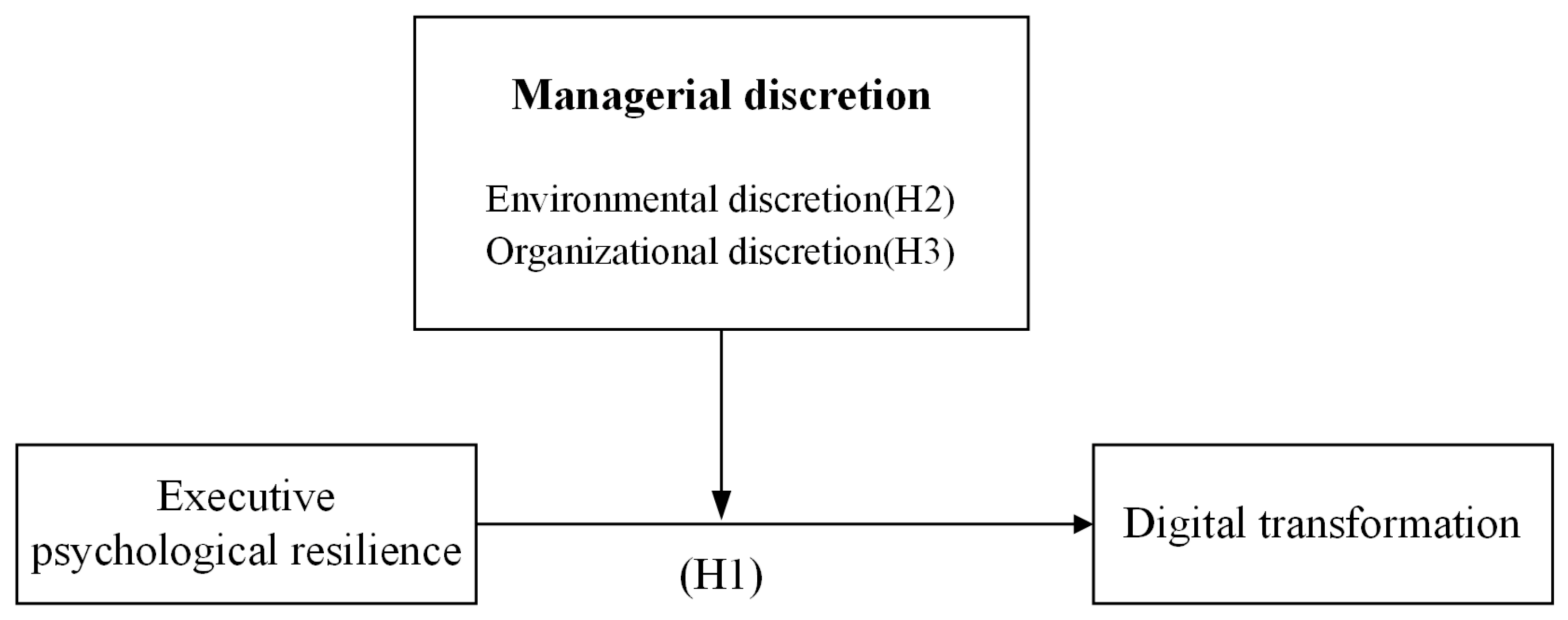
Theoretical framework.

Figure 2 illustrates the research framework of this study.

## 3. Methods

### 3.1. Sample and Data Source

Our initial sample includes Chinese A-share listed firms on the Shanghai and Shenzhen Stock Exchanges from 2011 to 2024. We apply the following screening procedures. First, we exclude firms designated as ST and ST*. Second, we eliminate listed enterprises with many missing values. Third, we exclude firms in the financial and insurance industries. Fourth, we excluded firms that had been established for fewer than five years. Finally, we winsorize all continuous variables at a 1% threshold to reduce the effect of potential spurious outliers (Barber & Lyon, 1996). The final sample comprises an unbalanced panel of 1979 listed firms with 23,843 firm-year observations. The firms in our sample span 74 two-digit industries based on the China Securities Regulatory Commission (CSRC) industry classification and are registered across 31 provincial-level administrative regions in mainland China.

### 3.2. Variable Measurement

#### 3.2.1. Dependent Variable: DT

We measure enterprise digital transformation using the enterprise digital transformation index provided by the China Stock Market and Accounting Research (CSMAR) database. The index was jointly developed by the CSMAR team and the “Intelligent Business and Innovative Enterprise Management” research group at East China Normal University. It is a weighted composite measure comprising six dimensions: strategic leadership, technological drive, organizational empowerment, environmental support, digital outcomes, and digital applications (Zhen et al., 2023). Accordingly, we adopt this composite index as the measure of enterprise digital transformation.

#### 3.2.2. Independent Variable: PR

Given the inherent challenges associated with obtaining direct psychological assessments from a large sample of executives, archival text-based approaches have become an increasingly common and valuable means of capturing managerial cognitive and psychological characteristics (Short et al., 2010). Language can reflect individuals’ cognition, preference and personality. Researchers capture human traits by analyzing word types and word frequencies used in languages (Pennebaker et al., 2003). For Chinese listed firms, the Management Discussion and Analysis (MD&A) section provides an executive’s review of the firm’s operating performance during the reporting period, as well as its discussion of future business plans, development opportunities, challenges, and potential risks. Consequently, using MD&A disclosures to capture an executive’s underlying cognition and personal traits has gained widespread acceptance among researchers, and the validity of this approach has been extensively supported in the literature. For example, Nadkarni and Barr (2008) conducted a textual analysis of Letters to Shareholders (LTS) from U.S. public firms and demonstrated how executive cognitive structures shape corporate strategic actions. Li (2010) utilized MD&A disclosures of U.S. listed firms to measure executive self-serving attribution bias. Therefore, textual disclosures in annual reports largely reflect the individual characteristics of the executive. In the Chinese context, the general manager (Zong jing li) or CEO performs a role largely comparable to that of a CEO in developed economies.

We adopt a text analysis approach based on the Management Discussion and Analysis (MD&A) sections of annual reports obtained from the Management Discussion and Analysis Database (CMDA) of the China Research Data Services Platform (CNRDS). Drawing on the managerial psychological resilience dictionary originally developed by McKenny et al. (2012) (provided in Appendix A), we employ the Chinese version developed and validated by Qiao et al. (2022). This dictionary was established through a rigorous process of translation, back-translation, and localization. We first conduct standard text preprocessing procedures, including the removal of punctuation marks, special symbols, and other non-textual content. Subsequently, we employ Python 3.13’s “Jieba” segmentation module to calculate the word frequencies of resilience-related words. To mitigate potential bias arising from differences in document length, we normalized the frequency by dividing the number of resilience-related words by the total number of words in each MD&A text and multiplying the result by 100. This normalized measure served as a proxy for executive psychological resilience.

#### 3.2.3. Moderating Variables

(1) Environmental discretion is measured by industry capital intensity, calculated as the average ratio of fixed assets to sales for firms within the same industry (Chen et al., 2025). (2) Organizational discretion is measured by slack resources, which are calculated as the average of unabsorbed slack and potential slack. Unabsorbed slack is measured by the current ratio (current assets divided by current liabilities), reflecting the availability of short-term liquid resources, whereas potential slack is measured by the equity-to-liabilities ratio (equity divided by liabilities), indicating a firm’s financial flexibility and borrowing capacity (Heubeck & Ahrens, 2024).

#### 3.2.4. Control Variables

Following existing work, we controlled for a set of variables at the firm, governance, and executive levels that may affect firms’ digital transformation. At the firm level, we controlled for firm size (size), firm age (age), state ownership (soe), profitability (roa), and revenue growth (growth). At the governance level, we controlled for ownership concentration (top1) and CEO duality (dual). At the executive level, we controlled for the proportion of male executives (male), managerial ownership (mor), and executives’ international experience (inter). In addition, we included firm fixed effects to control for time-invariant firm characteristics and province-year fixed effects to absorb time-varying shocks at the provincial level. Table 1 provides an overview of these controls and outlines their definitions.

### 3.3. Model Construction

We develop the below empirical models and employ Models (1), (2), and (3) to test Hypotheses (1), (2), and (3), respectively. In these models, *DT*_*i*,*t*_ is the dependent variable and represents the level of digital transformation of firm *i* in year *t*. *PR*_*i*,*t*_ is the independent variable and represents executive psychological resilience, and *PR*^2^_*i*,*t*_ denotes its squared term. *capi*_*i*,*t*_ represents industrial capital intensity. *slack*_*i*,*t*_ represents slack resources. *Controls*_*i*,*t*_ represents the control variables. *μ_i_* denotes firm fixed effects, *δ*_*p*,*t*_ denotes province-year fixed effects, and *ε*_*i*,*t*_ represents the error term.

(1) $$DT_{i,t}=\alpha_{0}+\alpha_{1}PR_{i,t}+\alpha_{2}{{PR}^{2}}_{i,t}+\gamma Controls_{i,t}+\mu_{i}+\delta_{p,t}+\varepsilon_{i,t}$$

(2) $$DT_{i,t}=\beta_{0}+\beta_{1}PR_{i,t}+\beta_{2}{{PR}^{2}}_{i,t}+\beta_{3}(PR_{i,t}\times capi_{i,t})+\beta_{4}({{PR}^{2}}_{i,t}\times capi_{i,t})+\beta_{5}capi_{i,t}+\gamma Controls_{i,t}+\mu_{i}+\delta_{p,t}+\varepsilon_{i,t}\gamma Controls_{i,t}+\mu_{i}+\delta_{p,t}+\varepsilon_{i,t}$$

(3) $$DT_{i,t}=\beta_{0}+\beta_{1}PR_{i,t}+\beta_{2}{{PR}^{2}}_{i,t}+\beta_{3}(PR_{i,t}\times slack_{i,t})+\beta_{4}({{PR}^{2}}_{i,t}\times slack_{i,t})+\beta_{5}slack_{i,t}+\gamma Controls_{i,t}+\mu_{i}+\delta_{p,t}+\varepsilon_{i,t}\gamma Controls_{i,t}+\mu_{i}+\delta_{p,t}+\varepsilon_{i,t}$$

## 4. Results

### 4.1. Validations of the Executive Psychological Resilience Measure

To assess the validity of the above text-based proxy for executive psychological resilience, we conducted three additional analyses. First, following Hassan et al. (2019), we estimate PR along several specifications and assess the *R*^2^ values to capture the portion of the variance of PR that is accounted for by fixed effects (see Appendix B). The results show that year and industry fixed effects explain only 8.06% and 12.03% of the variation in PR, respectively. Firm fixed effects account for 48.67% of the variation, suggesting that firms exhibit relatively stable disclosure patterns over time. Notably, executive fixed effects explain 63.64% of the variation in PR. In the stepwise regression analyses, after additionally controlling for industry, year, and firm fixed effects, the explanatory power of the model increases only marginally from 63.64% to 66.96%. Similar results are obtained when using adjusted R^2^. Thus, these findings suggest that the above text-based proxy captures meaningful executive-specific variation.

Moreover, we examine the relationship between the text-based proxy for executive psychological resilience and managerial tone (Tone). Although both measures are derived from the MD&A sections of annual reports, they are conceptually distinct. Tone, based on the Loughran and McDonald (2011) dictionary adapted to the Chinese context, captures the overall positivity or negativity of corporate disclosures, whereas the resilience dictionary focuses on language reflecting persistence, adaptation, recovery, and responses to adversity. The correlation between PR and Tone is 0.2562 (*p* < 0.001), indicating a relatively weak association. Furthermore, Tone explains only 6.56% of the variation in PR (R^2^ = 0.0656), indicating that the text-based proxy for executive psychological resilience derived from MD&A disclosures is empirically distinguishable from managerial disclosure tone.

Finally, we examine whether the text-based proxy for executive psychological resilience varies across different executive tenures within the same firm. If PR primarily reflects stable firm-level disclosure styles or communication patterns, it should remain relatively stable across different executives. In contrast, meaningful changes following executive turnover would suggest executive-specific characteristics. The executive turnover variable is constructed as follows. First, the executive for each firm-year is identified according to a hierarchical rule, with priority given to the chief executive officer, followed by the general manager. Next, the executive’s departure year is identified based on the recorded tenure end date. The executive turnover variable is coded as 1 if the identified executive leaves office during a given fiscal year and 0 otherwise. We calculate the average PR for each executive tenure and compare the absolute differences between consecutive executives within the same firm. The results show a mean (median) absolute difference of 0.786 (0.642), accounting for approximately 64.9% of the full-sample standard deviation of PR (1.211). These findings are consistent with the view that the above text-based proxy reflects executive-level heterogeneity beyond stable firm-level communication patterns (Hu et al., 2021).

### 4.2. Descriptive Statistics

The descriptive statistics for the variables are presented in Table 2. Both enterprise digital transformation and executive psychological resilience exhibit right-skewed distributions. This pattern is consistent with the current reality that, although some firms have made substantial progress in response to China’s continuous policy support for digital transformation, most firms remain at an early stage of digital development. Moreover, the mean value of executive psychological resilience exceeds its median value, further suggesting a right-skewed distribution characterized by a small number of firms with relatively high levels of executive psychological resilience. Furthermore, the VIF (variance inflation factor) values (Table 3) vary between 1.02 and 1.51, which is below the threshold of 5 set by (Hair et al., 2021). Therefore, the presence of multicollinearity is not deemed to be a concern.

### 4.3. Baseline

Based on the Hausman test, a fixed-effects model is employed to test the proposed hypotheses. To account for the potential correlation of error terms across observations from the same firm, we estimate cluster-robust standard errors at the firm level (Wooldridge, 2010). Table 4 presents the baseline regression results examining the relationship between executive psychological resilience and enterprise digital transformation. Column (1) presents the regression results including only control variables, and column (2) adds the linear term of executive psychological resilience, while column (3) further incorporates its quadratic term.

**Figure 3:**
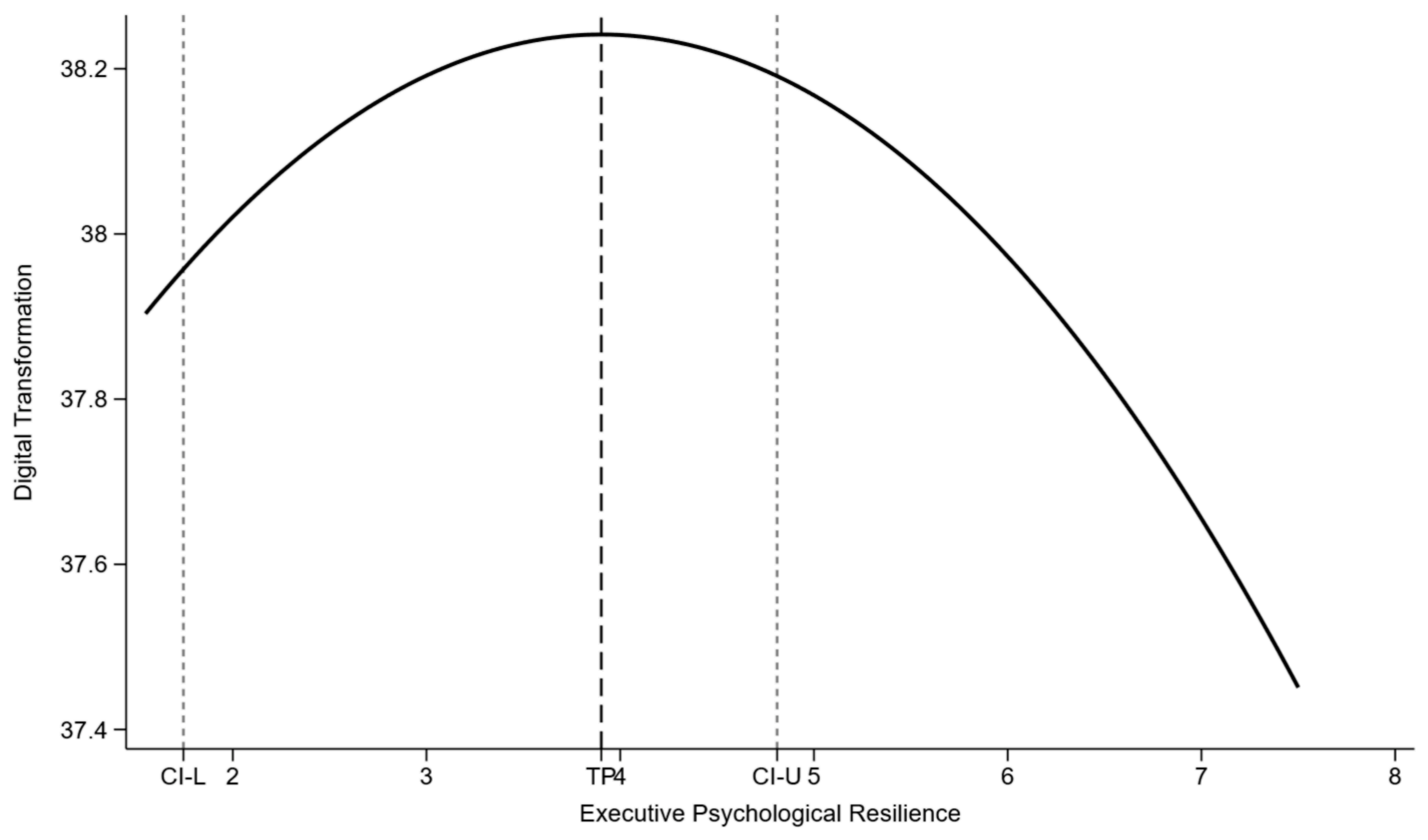
The inverted U-shaped relationship between executive PR and enterprise DT.

**Figure 4:**
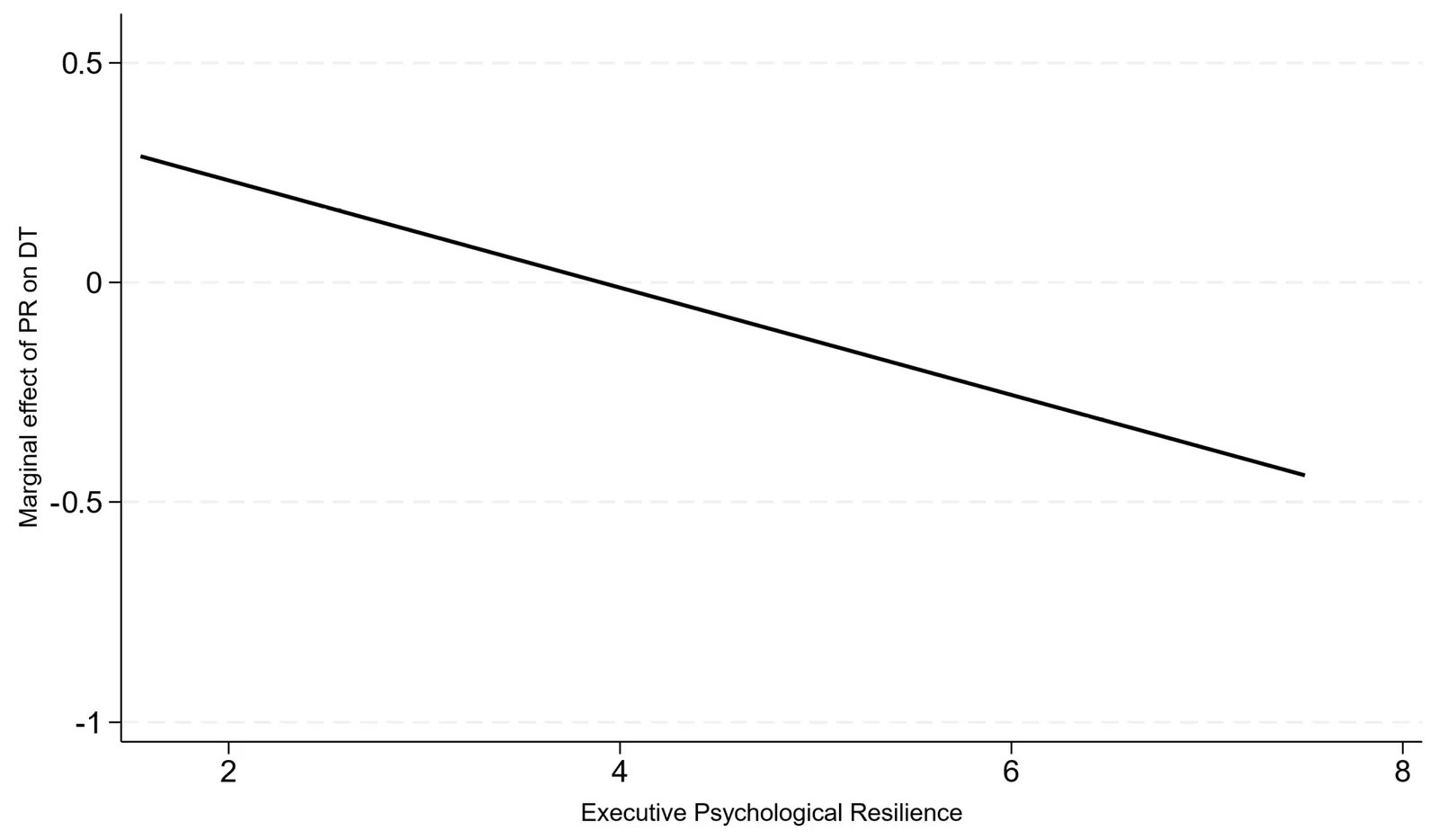
The marginal effect of executive PR on enterprise DT.

As shown in Column (2), the coefficient of executive psychological resilience is statistically insignificant, suggesting that its effect on digital transformation cannot be adequately captured by a simple linear relationship. The positive and negative effects associated with different levels of psychological resilience may offset each other, resulting in an insignificant average effect across the sample. Column (3) shows that the coefficient of the linear term of executive psychological resilience is positive (α_1_ = 0.477, *p* < 0.05), whereas the coefficient of the quadratic term is negative (α_2_ = −0.061, *p* < 0.05). The significance of these coefficients suggests an inverted U-shaped relationship between executive psychological resilience and enterprise digital transformation, as illustrated in Figure 3. Furthermore, the model including the quadratic term exhibits a higher within-R-squared value than the linear specification, indicating an improved model fit. In addition, we followed the three-step procedure proposed by Lind and Mehlum (2010) to further examine the inverted U-shaped relationship. As reported in Table 5, the observed range of executive psychological resilience is [1.55, 7.51], and the calculated turning point (−β_1_/2β_2)_ is 3.90. Furthermore, the 95% confidence interval for the turning point is [1.75, 4.81], which lies entirely within the observed range of executive psychological resilience. The slope at the lower bound of the interval is positive (0.28) and significant at the 5% level, whereas the slope at the upper bound is negative (−0.44) and significant at the 1% level. Taken together, these results provide evidence of an inverted U-shaped relationship between executive psychological resilience and enterprise digital transformation, thereby further supporting Hypothesis 1. Figure 4 further indicates the marginal effect of executive psychological resilience across different levels of resilience. As shown, the marginal effect declines with increasing levels of executive psychological resilience and becomes zero at the estimated turning point, where the total effect reaches its maximum. Beyond this point, the marginal effect becomes negative, indicating that further increases in executive psychological resilience reduce rather than enhance digital transformation.

### 4.4. Robustness Checks

(1) Introducing a cubic term. Following Haans et al. (2015), we introduced a cubic term of executive psychological resilience (X^3^) to examine whether the relationship is S-shaped rather than inverted U-shaped. As reported in column (1) of Table 6, the coefficients of the linear, quadratic, and cubic terms are all statistically insignificant. Therefore, there is no evidence supporting the existence of an S-shaped relationship, and the robustness of the previously identified inverted U-shaped relationship is further confirmed.

(2) Controlling for Industry–Year fixed effects. To further account for time-varying industry characteristics that may influence digital transformation, we additionally control industry-year fixed effects. The results are reported in column (2) of Table 6. The coefficient of the linear term of executive psychological resilience is significantly positive (β = 0.55, *p* < 0.01), whereas the coefficient of the squared term is significantly negative (β = −0.071, *p* < 0.01). The magnitude and statistical significance of the coefficients are even stronger than those in the baseline model, further reinforcing the robustness of the findings.

(3) Controlling for CEO turnover. We additionally control for CEO turnover because digital transformation is a long-term strategic process that may extend beyond the tenure of a single executive. Controlling for CEO turnover helps ensure that the observed relationship between executive psychological resilience and digital transformation is not merely driven by changes in executives (Saesen et al., 2024). The results reported in Column (3) of Table 6 show that the linear term of executive psychological resilience remains significantly positive, whereas the squared term remains significantly negative, both at the 5% significance level. This confirms the robustness of the inverted U-shaped relationship between executive psychological resilience and digital transformation after controlling for CEO turnover.

(4) Excluding Subsamples. First, given the potential economic disruptions caused by the 2015 Chinese stock market crash, which may have confounded the estimation results, we exclude observations from 2015 to 2016. Second, given that the COVID-19 outbreak in 2020 substantially affected firms’ business models and, consequently, their digital transformation processes, we exclude observations from 2020 to 2023 and re-estimate the model. As reported in column (4) of Table 6, the coefficient of the linear term of executive psychological resilience remains significantly positive, while the squared term remains significantly negative, indicating that the inverted U-shaped relationship between executive psychological resilience and enterprise digital transformation persists.

### 4.5. Endogeneity Assessment

**Figure 5:**
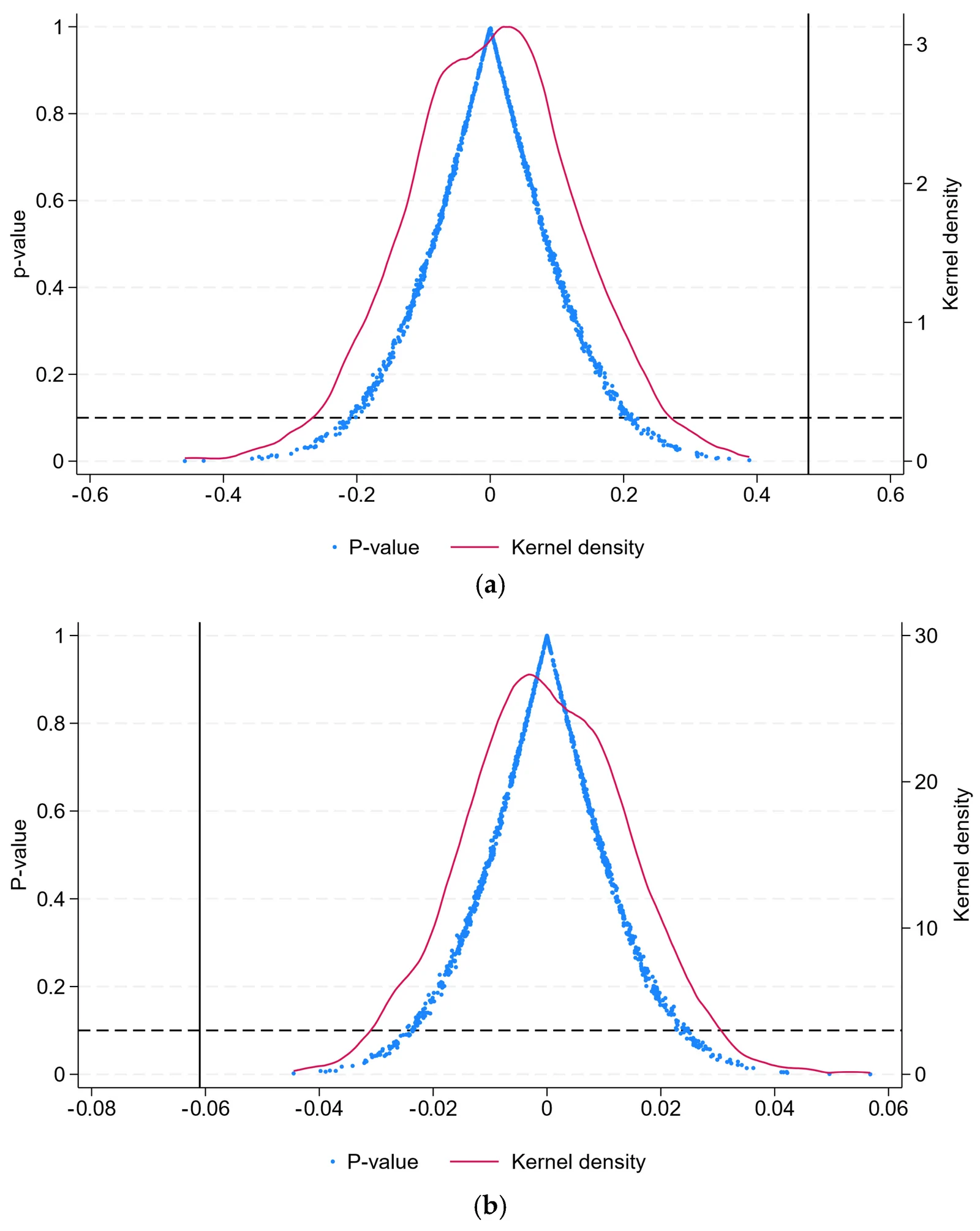
(**a**) placebo test for the linear term of executive psychological resilience. (**b**) placebo test for the squared term of executive psychological resilience.

We further address potential endogeneity concerns by employing lagged values of the key explanatory variables. Specifically, we re-estimate the baseline model using two-period lagged executive resilience as an alternative specification. This approach helps alleviate potential reverse causality between executive characteristics and firms’ current digital transformation decisions. As reported in Column (5) of Table 6, the results remain consistent with the baseline findings, suggesting the robustness of our main conclusions. Moreover, to alleviate concerns that the baseline results are driven by random factors, we conduct a placebo test. Specifically, we randomly permute the observed values of PR to construct a pseudo explanatory variable and re-estimate the baseline model. This permutation procedure is repeated 1000 times to obtain the empirical distribution of placebo estimates. As shown in Figure 5, the distributions of the placebo coefficients are centered around zero and exhibit no systematic deviation. Importantly, the actual baseline coefficients lie clearly outside the distributions of placebo estimates, with no meaningful overlap. These findings indicate that the observed inverted U-shaped relationship is unlikely to be driven by random variation. Overall, the placebo test provides further support for the robustness of our baseline results.

### 4.6. Moderation Analysis

#### 4.6.1. The Moderating Role of Industry Capital Intensity

**Figure 6:**
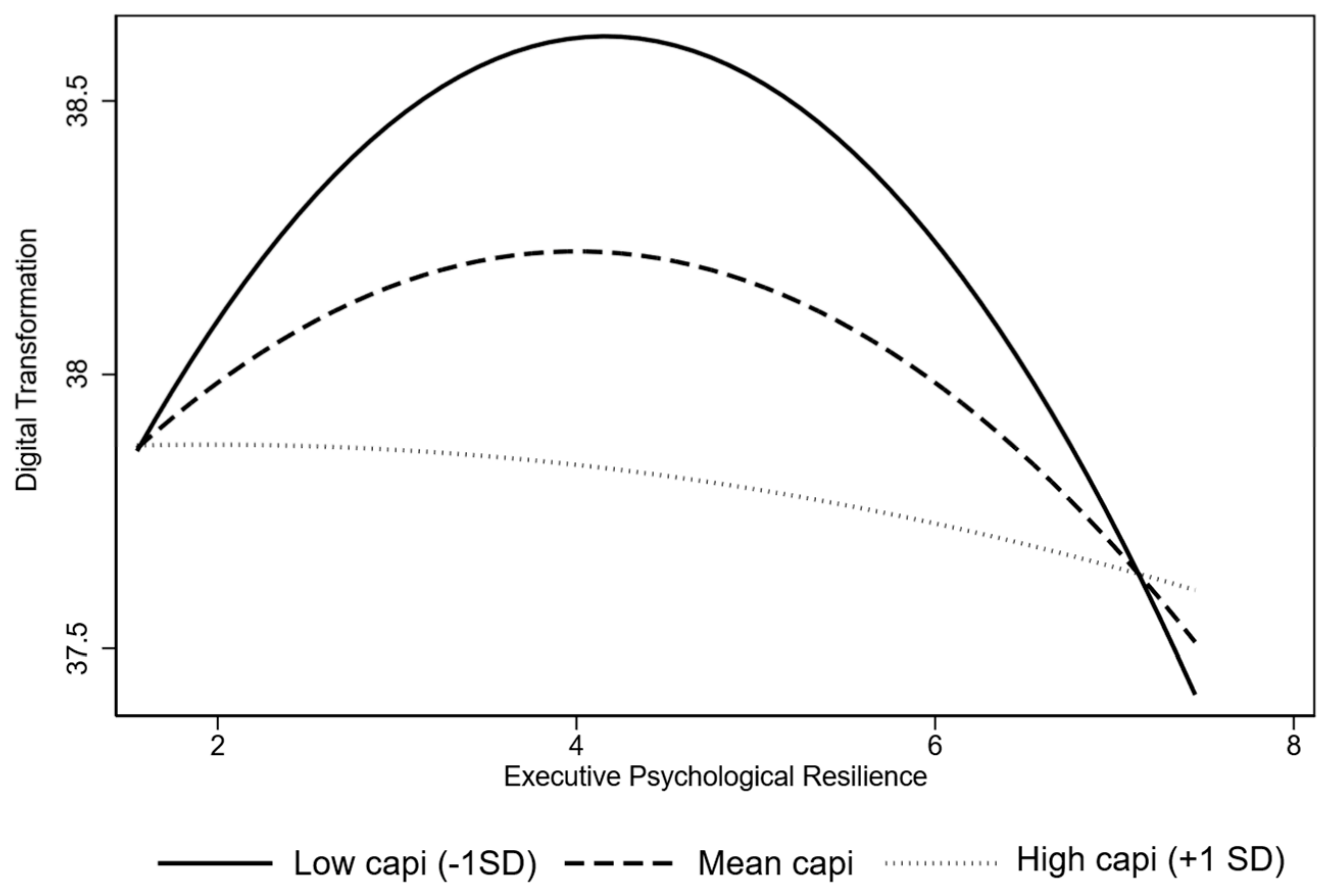
Relationship between PR and DT, moderated by industry capital intensity.

As reported in Column (4) of Table 4, the squared term of executive psychological resilience is significantly negative. More importantly, the interaction between industry capital intensity and the squared term of executive psychological resilience is significantly positive (β = 0.16, t = 2.77). These results indicate that industry capital intensity attenuates the inverted U-shaped relationship between executive psychological resilience and enterprise digital transformation. A possible explanation is that firms in capital-intensive industries are often constrained by substantial fixed-asset investments, which generate strong path dependence and limit strategic flexibility through prior resource commitments. As a result, executives possess less managerial discretion, reducing the extent to which their psychological resilience can influence enterprise digital transformation. These findings support Hypothesis 2. Consistent with the regression results, the moderation plot presented in Figure 6 further shows that the inverted U-shaped relationship becomes flatter under high levels of industry capital intensity than under low levels, indicating that the marginal effect of executive psychological resilience is weakened in more capital-intensive environments.

#### 4.6.2. The Moderating Role of Slack Resources

**Figure 7:**
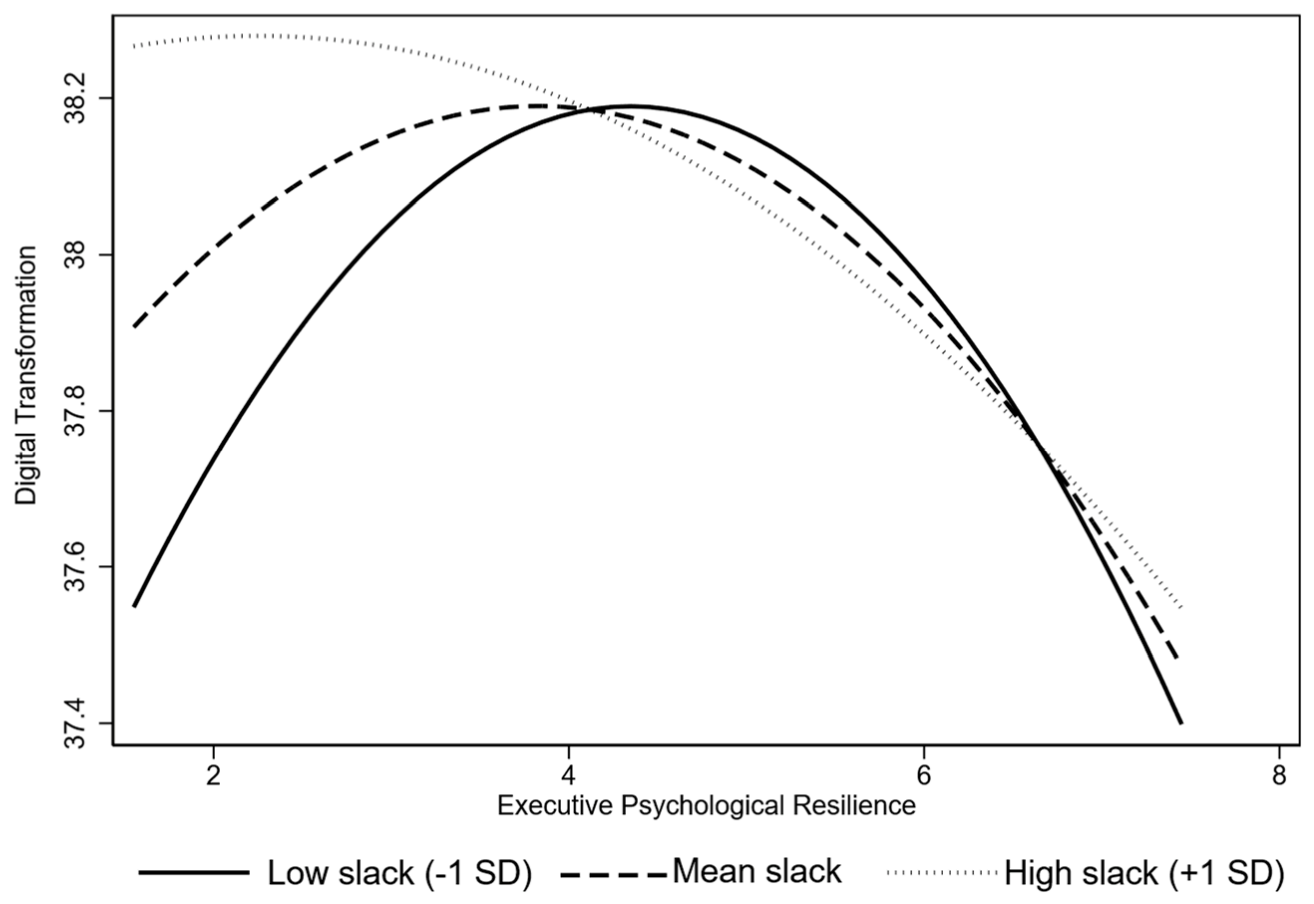
Relationship between PR and DT, moderated by slack resources.

According to Column (5) of Table 4, the coefficient of the interaction term between slack resources and the squared term of executive psychological resilience is not statistically significant, suggesting that slack resources do not significantly alter the curvature of the inverted U-shaped relationship. Therefore, Hypothesis 3 is not supported. However, following Haans et al. (2015), we further examine whether slack resources influence the location of the turning point. The estimated derivative of the turning point is negative and statistically significant at the 10% level (estimate = −0.0027, *p* = 0.065). The results suggest that slack resources shift the turning point of the inverted U-shaped relationship between executive psychological resilience and digital transformation leftward (see Figure 7). One possible explanation is that abundant slack resources provide firms with greater flexibility in allocating resources, allowing the benefits of executive psychological resilience for digital transformation to be realized at a lower level of resilience.

## 5. Discussion and Conclusions

Executives play a critical role in organizational success. In the context of DT, executives are in charge of setting the strategic direction of the business’ transformation and are responsible for guiding and coordinating the multiple organizational and operational changes resulting from the adoption of new digital technologies (Fernandez-Vidal et al., 2022). A traditional Chinese saying suggests that great responsibilities are often preceded by hardship and adversity. Therefore, Chinese executives tend to believe that higher levels of psychological resilience better equip them to confront challenges and lead their firms toward success. This cultural context provides a meaningful setting for examining the role of executive psychological resilience in digital transformation. Drawing on resource orchestration theory and the too-much-of-a-good-thing effect, we explore the relationship between executive psychological resilience and enterprise digital transformation and investigate the moderating effects of environmental discretion and organizational discretion. Based on empirical tests using data from A-share listed companies on Shanghai and Shenzhen stock exchanges from 2011 to 2024, the conclusions are as follows.

First, an inverted U-shaped association exists between executive psychological resilience and digital transformation. Consistent with upper echelons theory, executive psychological resilience is reflected in organizational strategic outcomes. Integrating resource orchestration theory, the results suggest that an optimal level of psychological resilience exists for digital transformation. Specifically, executives with higher psychological resilience tend to exhibit a stronger long-term orientation, greater self-efficacy and greater persistence in goal pursuit during the implementation of organizational tasks and activities (Santoro et al., 2020). These traits may enhance their willingness to promote resource orchestration action and digital transformation. However, beyond a certain threshold, executives may become overoptimistic and underestimate the risks associated with digital transformation. Meanwhile, the resulting strategic rigidity may weaken the effectiveness of resource orchestration and thereby hinder the transformation performance (Jiao et al., 2024; Williams et al., 2017). Importantly, although psychological resilience may produce similar effects to those of overconfidence and over-optimism once it exceeds a certain threshold, the three represent distinct constructs. Overoptimism and overconfidence are manifestations of systematic cognitive biases. Optimistic individuals tend to systematically overestimate the likelihood of favorable events while underestimating the likelihood of unfavorable ones. Overconfidence reflects excessive reliance on the accuracy of private information and an inflated belief in one’s own abilities. Accordingly, over-optimism reflects positive expectations about future outcomes, whereas overconfidence reflects excessive confidence in one’s own abilities and the accuracy of one’s judgments. Because both biases can lead to attributional distortions, they are often used interchangeably in the finance literature (Ataullah et al., 2018; Hilary et al., 2016). In contrast, psychological resilience emphasizes an individual’s capacity to recover, adapt, and continue functioning in the face of stress, setbacks, and uncertainty. When psychological resilience exceeds a certain threshold, individuals may develop biased interpretations of stress and setbacks, as well as distorted beliefs about their own capabilities and expected outcomes. This cognitive bias may ultimately influence decision-making and organizational outcomes. Second, this study places the associations between executive psychological resilience and digital transformation under two important sources of executive discretion, thus providing a more complex and nuanced understanding of their relationship. The influence of executive psychological traits on strategic change is contingent upon managerial discretion (Gao et al., 2024). The findings show that industry capital intensity negatively moderates this relationship by weakening the curvature of the inverted U-shaped relationship between executive psychological resilience and digital transformation. In contrast, the results provide some evidence that slack resources shift the turning point to the left, which may lead to an earlier onset of diminishing marginal effects. The findings are consistent with prior research, suggesting that the psychological traits of top executives do not directly translate into firm-level behaviors but are instead contingent upon managerial discretion shaped by both industry-level environmental conditions and firm-level organizational contexts (Kiss et al., 2024; Saeed et al., 2024).

## 6. Implications

This study makes two primary contributions. First, existing studies have primarily focused on observable executive characteristics, such as demographic background, career experience, and digital capabilities (Yan et al., 2023). Some studies have also explored how executives’ implicit cognitive structures and overconfidence influence enterprise digital transformation (Saesen et al., 2024; Zhu et al., 2022). However, these factors provide limited insight into how executives cope with uncertainty during digital transformation and recover from transformation setbacks. By incorporating executive psychological resilience into digital transformation research, this study draws on resource orchestration theory to provide insights into how executive psychological resilience may be reflected in resource structuring, resource bundling, and resource leveraging behaviors, as well as executives’ responses to challenges such as organizational inertia and short-term performance declines during the digital transformation process. Second, this study advances the literature on executive psychological resilience by examining its nonlinear effect on enterprise digital transformation. Executives facilitate corporate digital transformation by understanding digitalization, setting the formal context for digitalization, and leading organizational change in response to the challenges brought by the digital wave (Wrede et al., 2020). While considerable attention has been devoted to the positive effects of executive characteristics on digital transformation, an increasing number of studies have begun to acknowledge executives’ bounded rationality in strategic decision-making (Saesen et al., 2024; Xu et al., 2025). Our findings show that excessive resilience may also hinder digital transformation, suggesting that the benefits of executive psychological resilience are not unlimited. By identifying the threshold at which resilience shifts from being beneficial to potentially detrimental, this study responds to calls for adopting a nonlinear perspective when examining relationships among constructs in management research. Furthermore, this study contributes to emerging research on executive bounded rationality by offering a more nuanced understanding of how executives’ personal traits are associated with firms’ strategic transformation.

For practitioners, our findings provide several implications for managing digital transformation. First, the results highlight a double-edged sword effect of executive psychological resilience in shaping digital transformation outcomes. Our research offers guidance for board members involved in the CEO selection. Digital transformation involves complex and high-stakes decisions that require forward-looking leadership. Accordingly, organizations should consider not only executives’ experience and expertise but also whether their psychological characteristics fit the firm’s strategic objectives and operating environment. Firms may benefit from paying greater attention to psychological resilience when selecting and developing executives and from recognizing both its potential benefits and limitations in strategic decision-making. At the same time, firms should remain mindful of how internal systems—such as slack resources—and external conditions might either amplify or mitigate the influence of executive psychological resilience. Second, for executives immersed in the profound turbulence of digital transformation, this finding suggests the value of balancing perseverance with timely reassessment. When businesS-side feedback remains unfavorable for a prolonged period or when the costs brought by digitalization continue to exceed expectations, executives should slow down the pace to conduct small-scale loss containment rather than blindly implementing aggressive strategies.

## 7. Limitations and Future Research

This study has several limitations that provide avenues for future research. First, psychological resilience is a dynamic psychological capacity that may evolve over time in response to environmental shocks and managerial experiences. However, due to the constraints of panel data, our measurement approach cannot directly capture the formation and evolutionary process of resilience. Future studies may adopt real—time tracking data or longitudinal psychological assessments to better capture the dynamic nature of executive resilience. Furthermore, while textual analysis offers a scalable and widely used approach to measuring executive psychological resilience, it may be subject to impression management and disclosure practices. Future research could combine textual indicators with survey-based measures, interviews, or other complementary data sources to further validate and refine the measurement of executive psychological resilience. Second, given the inherent complexity of individual psychological traits and data limitations, this study does not examine the underlying mechanisms through which executive psychological resilience influences firms’ digital transformation. Future research is encouraged to further explore and test these mediating pathways. Moreover, this study focuses solely on individual executive resilience while overlooking its peer effects. Future research could further examine how the resilience of top management teams influences enterprise digital transformation. Finally, our sample is limited to Chinese A—share listed firms, which may constrain the generalizability of the findings. Future research could extend the analysis to non-listed firms or firms operating in different national contexts to assess the applicability of the findings across different organizational and institutional settings.

## Figures and Tables

**Table 1:** Control variable definitions.

Variable Name	Code	Variable Definitions
firm size	size	Natural logarithm of total assets
firm age	age	The observation year minus the year of the firm’s incorporation
nature of property rights	soe	A dummy variable that takes the value of 1 if the ultimate controller is state-owned, and 0 otherwise
profitability	roa	Net profit divided by the average balance of total assets
operating revenue growth rate	growth	(Current year’s operating revenue—Previous year’s operating revenue)/Previous year’s operating revenue
ownership concentration	top1	Shareholding percentage of the largest shareholder
CEO duality	dual	A dummy variable that takes the value of 1 if the Chairman and CEO are the same person, and 0 otherwise
proportion of male executives	male	The proportion of male members in the top management team
executives’ international experience	inter	A dummy variable that takes the value of 1 if any director, supervisor, or senior executive has an overseas background, and 0 otherwise
managerial ownership	mor	Percentage of shares held by the management

**Table 2:** Descriptive statistics.

Variables	N	Mean	SD	Min	Median	Max
DT	23,842	38.035	10.595	23.276	36.275	64.968
PR	22,588	4.034	1.211	1.550	3.930	7.510
capi	23,841	0.358	0.319	0.009	0.282	3.461
slack	23,212	2.508	2.563	0.378	1.620	15.910
size	23,843	22.309	1.292	20.151	22.112	26.386
age	23,842	19.370	6.020	7.000	19.000	36.000
soe	23367	0.317	0.465	0.000	0.000	1.000
roa	23,843	0.042	0.055	−0.177	0.040	0.194
growth	23,839	0.134	0.288	−0.486	0.097	1.554
dual	23498	0.302	0.459	0.000	0.000	1.000
top1	23,842	33.384	14.536	8.440	31.270	74.660
male	23,843	80.015	11.402	47.060	81.250	100.000
mor	23,418	15.696	20.024	0.000	3.640	69.705
inter	23,843	0.605	0.489	1.000	0.000	1.000

**Table 3:** Bivariate correlation matrix.

Variables	(1)	(2)	(3)	(4)	(5)	(6)	(7)	(8)	(9)	(10)	(11)	(12)
(1) DT	1.000											
(2) PR	0.202 *	1.000										
(3) size	0.077 *	−0.066 *	1.000									
(4) age	0.094 *	0.035 *	0.283 *	1.000								
(5) soe	−0.080 *	−0.120 *	0.385 *	0.196 *	1.000							
(6) roa	−0.105 *	0.045 *	−0.023 *	−0.134 *	−0.082 *	1.000						
(7) growth	−0.004	0.012	−0.012	−0.137 *	−0.077 *	0.285 *	1.000					
(8) dual	0.069 *	0.073 *	−0.190 *	−0.089 *	−0.302 *	0.026 *	0.034 *	1.000				
(9) top1	−0.184 *	−0.079 *	0.161 *	−0.120 *	0.206 *	0.151 *	0.008	−0.045 *	1.000			
(10) male	−0.080 *	−0.052 *	0.166 *	−0.058 *	0.219 *	0.009	0.021 *	−0.125 *	0.066 *	1.000		
(11) mor	0.003	0.101 *	−0.386 *	−0.242 *	−0.488 *	0.143 *	0.100 *	0.233 *	−0.084 *	−0.159 *	1.000	
(12) inter	0.105 *	0.043 *	0.066 *	−0.006	−0.092 *	0.014 *	0.024 *	0.018 *	−0.035 *	−0.016 *	0.021 *	1.000
VIF		1.03	1.34	1.21	1.51	1.15	1.11	1.12	1.11	1.07	1.45	1.02

Note: * represents statistical significance at 10%.

**Table 4:** Baseline and moderating effect regression results.

Variables	(1) DT	(2) DT	(3) DT	(4) DT	(5) DT
PR		−0.056	0.477 **	0.979 ***	0.711 **
		(−1.088)	(2.216)	(3.293)	(2.454)
PR^2^			−0.061 **	−0.117 ***	−0.076 **
			(−2.517)	(−3.436)	(−2.275)
capi				1.793 *	
				(1.819)	
PR × capi				−1.395 ***	
				(−2.986)	
PR^2^ × capi				0.160 ***	
				(2.771)	
slack					0.344 **
					(2.068)
PR × slack					−0.125
					(−1.616)
PR^2^ × slack					0.010
					(1.070)
size	1.520 ***	1.519 ***	1.521 ***	1.520 ***	1.488 ***
	(8.345)	(8.324)	(8.336)	(8.344)	(7.908)
age	−0.077	−0.082	−0.080	−0.077	−0.103
	(−0.443)	(−0.468)	(−0.462)	(−0.442)	(−0.575)
soe	−0.678 *	−0.686 *	−0.679 *	−0.672 *	−0.643
	(−1.712)	(−1.736)	(−1.724)	(−1.709)	(−1.598)
roa	−1.541	−1.505	−1.515	−1.684 *	−1.676
	(−1.534)	(−1.500)	(−1.511)	(−1.676)	(−1.610)
growth	0.020	0.018	0.015	0.008	0.012
	(0.173)	(0.150)	(0.131)	(0.067)	(0.101)
dual	0.276 *	0.278 *	0.275 *	0.269 *	0.255
	(1.719)	(1.729)	(1.710)	(1.681)	(1.557)
top1	−0.011	−0.011	−0.011	−0.011	−0.010
	(−0.929)	(−0.913)	(−0.929)	(−0.884)	(−0.805)
male	0.008	0.008	0.008	0.008	0.009
	(1.010)	(1.022)	(1.047)	(1.002)	(1.066)
mor	0.005	0.005	0.005	0.005	0.004
	(0.638)	(0.694)	(0.642)	(0.639)	(0.473)
inter	0.207	0.206	0.207	0.208	0.198
	(1.577)	(1.565)	(1.574)	(1.589)	(1.490)
Firm/Province-Year FE	YES	YES	YES	YES	YES
Observations	21,435	21,435	21,435	21,433	20,861
Within R-squared	0.0202	0.0203	0.0210	0.0221	0.0208

Notes: t-statistics in parentheses. Standard errors are clustered at the firm level to account for non-independence of errors. *** *p* < 0.01, ** *p* < 0.05, * *p* < 0.10. The same notation applies to all subsequent tables.

**Table 5:** Results of the U test.

Specification: F(X) = X^2^		
PR	α_1_	0.477
PR^2^	α_2_	−0.061
Extreme point	−α_1_/2α_2_	3.90
Slope at X_L_	α_1_ + 2α_2_X_L_	0.28 **
		(2.00)
Slope at X_H_	α_1_ + 2α_2_X_H_	−0.44 ***
		(−2.69)
95% Fieller interval for extreme point	[1.75, 4.80]	
Overall test of presence of an Inverse U shape	t-value	2.01
	*p*-value	0.02

Notes: *** *p* < 0.01, ** *p* < 0.05.

**Table 6:** Results of robustness checks.

Variables	(1) DT	(2) DT	(3) DT	(4) DT	(5) DT
PR	0.24	0.55 ***	0.47 **	0.59 **	0.31
	(0.33)	(2.69)	(2.20)	(2.28)	(1.57)
PR^2^	−0.003	−0.071 ***	−0.006 **	−0.07 **	−0.05 **
	(−0.02)	(−3.09)	(−2.49)	(−2.36)	(−2.23)
PR^3^	−0.004				
	(−0.35)				
Controls	YES	YES	YES	YES	YES
Firm/Province-Year FE	YES	YES	YES	YES	YES
Industry-Year FE	NO	YES	NO	NO	NO
Observations	21,435	21,353	21,410	14,996	17,634
Within R-squared	0.0210	0.0188	0.0211	0.0210	0.0173

Notes: *** *p* < 0.01, ** *p* < 0.05.

## Data Availability

The data used to support the findings of this study are available from the corresponding author upon request.

## Appendix A

**Table A1:** Resilience dictionary.

Adamant, Adamantly, Assiduous, Assiduously, Assiduousness, Backbone, Bandwidth, Bears up, Bounce, Bounced, Bounces, Bouncing, Buoyant, Commitment, Commitments, Committed, Consistent, Determination, Determined, Determinedly, Determinedness, Devoted, Devotedly, Devotedness, Devotion, Die trying, Died trying, Dies trying, Disciplined, Dogged, Doggedly, Doggedness, Drudge, Drudged, Drudges, Endurance, Endure, Endured, Endures, Enduring, Grit, Hammer away, Hammered away, Hammering away, Hammers away, Held fast, Held good, Held up, Hold fast, Holding fast, Holding up, Holds fast, Holds good, Immovability, Immovable, Immovably, Indefatigable, Indefatigableness, Indefatigably, Indestructibility, Indestructible, Indestructibleness, Indestructibly, Intransigence, Intransigency, Intransigent, Keep at, Keep going, Keep on, Keeping at, Keeping going, Keeping on, Keeps at, Keeps going, Keeps on, Kept at, Kept going, Kept on, Labored, Laboring, Never-tiring, Never-wearying, Perdure, Perdured, Per during, Perseverance, Persevere, Persevered, Persevering, Persist, Persisted, Persistence, Persistent, Persisting, Pertinacious, Pertinaciously, Pertinacity, Rebound, Rebounded, Rebounding, Rebounds, Relentlessness, Remain, Remained, Remaining, Remains, Resilience, Resiliency, Resilient, Resolute, Resolutely, Resoluteness, Resolve, Resolved, Resolves, Resolving, Robust, Sedulity, Sedulous, Sedulously, Sedulousness, Snap back, Snapped back, Snapping back, Snaps back, Spring back, Springing back, Springs, Springs back, Sprung back, Stalwart, Stalwartly, Stalwartness, Stand fast, Stand firm, Standing fast, Standing firm, Stands fast, Stands firm, Stay, Steadfast, Steadfastly, Steadfastness, Stood fast, Stood firm, Strove, Survive, Surviving, Surviving, Tenacious, Tenaciously, Tenaciousness, Tenacity, Tough, Uncompromising, Uncompromisingly, Uncompromisingness, Unfaltering, Unfalteringly, Unflagging, Unrelenting, Unrelentingly, Unrelentingness, Unshakable, Unshakablely, Unshakeable, Unshaken, Unshaking, Unswervable, Unswerved, Unswerving, Unswervingly, Unswervingness, Untiring, Unwavered, Unwavering, Unweariedness, Unyielding, Unyieldingly, Unyieldingness, Upheld, Uphold, Upholding, Upholds, Zeal, Zealous, Zealously, Zealousness

## Appendix B

**Table A2:** Variance decomposition analysis.

	PR Text Measure (R-Squared)	PR Text Measure (Adjusted R-Squared)
Univariate Regression		
Year Fixed Effect	0.0806	0.0801
Industry Fixed Effect	0.1203	0.1174
Firm Fixed Effect	0.4867	0.4365
Manager Fixed Effect	0.6364	0.5521
Stepwise Regression		
Manager Fixed Effect	0.6500	0.5637
+Year Fixed Effect	0.6647	0.5865
+Industry Fixed Effect	0.6680	0.5890
